# Antimicrobial and Anti-Inflammatory Activities of *Pterygota macrocarpa* and *Cola gigantea* (Sterculiaceae)

**DOI:** 10.1155/2012/902394

**Published:** 2012-05-28

**Authors:** Christian Agyare, George Asumeng Koffuor, Vivian Etsiapa Boamah, Francis Adu, Kwesi Boadu Mensah, Louis Adu-Amoah

**Affiliations:** ^1^Department of Pharmaceutics, Faculty of Pharmacy and Pharmaceutical Sciences, Kwame Nkrumah University of Science and Technology, Kumasi, Ghana; ^2^Department of Pharmacology, Faculty of Pharmacy and Pharmaceutical Sciences, Kwame Nkrumah University of Science and Technology, Kumasi, Ghana

## Abstract

*Pterygota macrocarpa* and *Cola gigantea* are African medicinal plants used in traditional medicine for the treatment of sores, skin infections, and other inflammatory conditions including pains. This study therefore aims at investigating the antimicrobial properties of ethanol leaf and stem bark extracts of *P. macrocarpa* and *C. gigantea* using the agar diffusion and the micro-dilution techniques and also determining the anti-inflammatory properties of the extracts of these plants in carrageenan-induced foot edema in seven-day old chicks. The minimum inhibitory concentration of both ethanol leaf and bark extracts of *P. macrocarpa* against the test organisms was from 0.125 to 2.55 mg/mL and that of *C. gigantea* extracts was 0.125 to 2.75 mg/mL. Extracts with concentration of 50 mg/mL were most active against the test organisms according to the agar diffusion method. All the extracts of *P. macrocarpa* and *C. gigantea* at 30, 100, and 300 mg/kg body weight except ethanol leaf extract of *C. gigantea* exhibited significant anti-inflammatory effects (*P* ≤ 0.001).

## 1. Introduction

The search for newer antimicrobial agents from various sources has become imperative because of the emergence of resistance strains of microorganisms against orthodox antibiotics especially difficulty to treat infections from resistant strains of bacteria [1] and also the fact that the number of scientists who are developing new antibacterial agents has dwindled, even as bacteria evolve ever more clever mechanisms of resistance to antibiotics [2]. The recent search for new antibiotics includes various sources such as the synthetic compounds, bioactive agents from aquatic microorganisms, and natural products including medicinal plants. In Africa and other developing countries, it is estimated that 70 to 80% of people rely on traditional healers and herbal practitioners for their health needs [3, 4] and medicinal plants are the main source of remedies used in this therapy. Some of these medicinal plants are used for the management of several different disease conditions such as bacterial infections, parasitic infections, skin diseases, hypertension, pains, and inflammation such as rheumatoid arthritis [5–8].

Several medicinal plants including their isolated compounds have been found to exhibit biological activities related to their traditional uses, for example, geraniin and furosin isolated from *Phyllanthus mellerianus* (Kuntze) Exell. have been found to possess wound healing properties ascribed to this plant as wound healing agent [9]. Cryptolepine, an alkaloid from *Cryptolepine sanguinolenta*, has been shown to possess antimicrobial and antiplasmodial activities which have gone to confirm its medicinal uses as anti-infective and antimalarial agent [10, 11].

*Pterygota macrocarpa* K. Schum. belongs to the family Sterculiaceae and is known in local Asante-Twi language as *kyereye* in Ghana. It is a large tree that grows in dense semideciduous forests usually distributed in West Africa from Sierra Leone to Cameroun. The soaked leaves are used to treat stomachache, pains, and disorders of digestion. Leaf decoctions are used for the treatment of gonorrhea and other urinary tract infections [12–14]. Traditionally, the bark is used in the management of haemorrhoids, dropsy, swellings, edema, gout, leprosy, and pain [15]. The seeds of *P. macrocarpa *have been found to contain phytate, oxalate and tannins [16].

*Cola gigantea* A. Chev. belongs to the family Sterculiaceae and is commonly known as giant cola and local *Asante-Twi* name is *watapuo* in Ghana. It is a large tree in dry semideciduous forests in West Africa and the West Indies. The nuts (mostly called kola) are often used to treat whooping cough, asthma, malaria, and fever. Other traditional uses include increasing the capacity for physical exertion and for enduring fatigue without food, stimulating a weak heart, and treating nervous debility, weakness, lack of emotion, nervous diarrhea, depression, despondency, brooding, anxiety, and sea sickness [12, 14, 15, 17]. Kola nut is the name of the mature fruits of the *Cola *species [18] and has a bitter flavour and high caffeine content [19, 20], and when the fruit is ingested, it acts as stimulants and thus creates an ecstatic and euphoric state [20]. The caffeine present acts as a bronchodilator, expanding the bronchial air passages [21]. These fruits are also chewed in communities during traditional ceremonies and also are known to reduce hunger pangs. The ethanol leaf extract of *C. gigantea* has been shown to be active against *Candida albicans* and phytochemical screening of the leaf extract indicated the presence of alkaloids, saponins, tannins, anthraquinones, and cardenolides [22]. The aim of this study is to investigate the antimicrobial and anti-inflammatory activities of ethanol stem bark and leaf extracts of *P. macrocarpa *and *C. gigantea*.

## 2. Materials and Methods

### 2.1. Plant Material and Chemicals

Stem bark and leaves of *Pterygota macrocarpa* and *Cola gigantea *were collected in July, 2007, from the Bobiri Forest Reserves of the Forestry Research Institute of Ghana (FORIG) near Kubease, Ashanti Region, Ghana, and identified and authenticated by Dr. A. Asase, Department of Botany, University of Ghana, Ghana. Unless stated otherwise, all the chemicals were purchased from Sigma (Deisenhofen, Germany).

### 2.2. Preparation of Extracts

The plant materials were air dried and powdered, and 200 g each of the dried powdered material of *P. macrocarpa* and *C. gigantea *was extracted, respectively, with 70% ethanol (1.5 L) using Soxhlet apparatus. The ethanol extracts obtained were evaporated to dryness under reduced pressure and kept in a dessicator. The yields of the stem and leaf extracts of *P. macrocarpa* were 4.2 and 12.4% w/w, respectively. And the yields of *C. gigantea* were 3.6 and 16.5% w/w for stem bark and leaf extracts, respectively. Various quantities of the ethanol leaf extract (CGLE) and ethanol stem bark extract (CGBE) of *C. gigantea *and ethanol leaf extract (PMLE) and ethanol stem bark extract (PMBE) of *P. macrocarpa *were dissolved in normal saline and methanol for acute anti-inflammatory and antimicrobial determinations, respectively.

### 2.3. Preliminary Phytochemical Screening

Phytochemical screening was conducted on both leaf and stem bark of *P. macrocarpa* and *C. gigantea* to ascertain the presence of carbohydrates, tannins, sapogenetic glycosides, flavonoids, steroids, and alkaloids [23, 24]. The tannins content was determined according to the method of Glasl [25] using pyrogallol (Merck, Darmstadt, Germany, purity 99.5%, HPLC) as reference compound.

### 2.4. Determination of Antimicrobial Activity

#### 2.4.1. Agar Diffusion Method

The antimicrobial activities of the extracts (PMLE, CGLE, PMBE, and CGBE) and reference drugs (chloramphenicol and clotrimazole (Sigma, Deisenhofen, Germany)) were determined using the agar diffusion method [26]. Nutrient agar (Oxoid Limited, United Kingdom) and Sabouraud agar (Oxoid Limited, United Kingdom) media were used for both determinations of antibacterial and antifungal activities, respectively. 0.1 mL of 18 h culture of the test organisms (*Escherichia coli *ATCC 25922, *Pseudomonas aeruginosa *ATCC 27853, *Staphylococcus aureus* ATCC 25923, *Bacillus subtilis *NCTC 10073, and clinical fungal agent, *Candida albicans, *were used to seed nutrient agar and Sabouraud agar plates, respectively. In each of these plates, 4 equidistant wells (10 mm) were cut out using sterile cork borer and were filled with 200 *μ*L each of the different concentrations of extracts and reference drugs and allowed to diffuse at room temperature for 1 h. The zones of inhibition were measured after 24 h incubation at 37°C (for bacteria) and after 72 h at 30°C (for fungi). The activities of the methanol (solvent) alone were also determined.

### 2.5. Determination of Minimum Inhibitory Concentration (MIC) Using Microdilution Technique

The MICs of the extracts (PMLE, CGLE, PMBE, and CGBE) against the test bacteria were determined using the microdilution technique as described by Eloff [27] and modified by Agyare and Koffuor [26]. Test solutions (100 mg/mL) of both extracts were prepared, test solution (25–100 *μ*L) was serially diluted with distilled water to 100 *μ*g/mL, and 50 *μ*L of an 18 h old culture of one of the test bacteria grown in nutrient broth (Oxoid Limited, United Kingdom) was added to each well in the microplates. The covered microplates were incubated at 37°C for 24 h. To indicate growth, 30 *μ*L of 3-(4,5-dimethylthiazol-2-yl)-2,5-diphenyltetrazolium bromide (MTT, thiazolyl blue) dissolved in distilled water was added to the microplate wells and incubated at 37°C for 30 min. *C. albicans* was cultivated in Sabouraud dextrose broth (Oxoid Limited, United Kingdom) and then incubated for 3 days at 30°C. The MICs of PMLE, CGLE, PMBE, and CGBE against the test fungus were determined according to the guidelines described in the National Committee for Clinical Laboratory Standards [28] for filamentous fungi. The minimum inhibitory concentration of the each extract against the test organisms was detected as the minimum concentration of extracts where there was no microbial growth, that is, nonformation of blue color after the addition MTT to the medium [29]. The previous experiment was repeated three times.

### 2.6. Determination of Acute Anti-Inflammatory Activity

The carrageenan-induced inflammatory model in seven-day-old chicks [30] was employed and the responsiveness of these chicks to anti-inflammatory drugs/extracts was determined.

### 2.7. Experimental Animals

7-days-old cockerels *Gallus gallus* (100–120 g) (Strain: Shaver 579) purchased from Darko Farms Company Limited, Kumasi, Ghana, were maintained in the Animal House of the Department of Pharmacology, Kwame Nkrumah University of Science and Technology, Kumasi, Ghana. The chicks were housed in stainless steel cages and fed with normal commercial poultry diet (GAFCO, Tema, Ghana), given water *ad libitum,* and maintained under laboratory conditions (temperature 28–30°C, relative humidity 60–70%, and normal light-dark cycle). A day before the experiment, the chicks were brought to the laboratory and habituated to experimenter handling and the apparatus to minimize the effect of stress and novelty. The 7-day-old chicks were used in experiment. All procedures and techniques used in these studies were in accordance with the National Institute of Health Guidelines for the Care and Use of Laboratory Animals (NIH, Department of Health Services publication no. 83-23, revised 1985). The protocols for the study were approved by the Departmental Ethics Committee.

### 2.8. Experimental Design

At the beginning of the experiment the chicks (7 days old) were randomly assigned to one of fifteen groups (*n* = 5). The initial foot volumes of the chicks were measured using plethysmometer (IITC Life Science Inc., CA, USA) after which 0.01 mL of 2% carrageenan was injected into the plantar of the right foot to induce inflammation. The inflammation produced was measured, the increase in foot volumes was calculated, and those with an increase between 15 and 40% were selected and put into thirteen groups of five after which they were injected intraperitoneally with either diclofenac (Sigma, purity 98% HPLC) (10, 30 and 100 mg/kg) or dexamethasone (Sigma, purity 98% HPLC) (0.25, 0.5, and 1.0 mg/kg) based on recommended effective human doses per body weight and PMLE, CGLE (30, 100, and 300 mg/kg), or PMBE and CGBE (30, 100 and 300 mg/kg) given orally based on preliminary investigation. One group did not receive any drug (control). Foot volumes were measured again at hourly interval posttreatment for 4 h. The percentage change in foot volume after induction and treatment of inflammation was calculated and recorded for analysis.

### 2.9. Statistical Analysis

GraphPad Prism Version 5.0 for Windows (GraphPad Software, San Diego, CA, USA) was used for all statistical analyses. Data are presented as mean ± SEM (*N* = 5) and analyzed by one-way ANOVA followed by Dunnett's multiple comparison's test. *P* < 0.05 was considered statistically significant in all analyses. The graphs were plotted using Sigma Plot for Windows version 11.0 (Systat Software Inc., Germany).

## 3. Results

### 3.1. Preliminary Phytochemical Screening

Both the leaf and stem bark of *P. macrocarpa* and *C. gigantea* were found to contain tannins (with varying amounts), alkaloids, steroids, saponins, and carbohydrate while the leaves of the two plants contain flavonoids. The stem bark and leaves of *P. macrocarpa* were found to contain sapogenetic glycosides (Table 1).

### 3.2. Antimicrobial Activity

The ethanol extracts (PMLE, PMBE, CGLE, and CGBE) were found to be active against the test bacteria (*E. coli*, *P. aeruginosa*, *S. aureus, B. subtilis*) with varying mean zones of inhibition and* C. albicans *was found to be less susceptible to the extracts. With respect to the agar diffusion method, the extracts (PMLE, CGLE, PMBE, and CGBE) with concentrations of 50 mg/mL exhibited the highest activity against the test organisms (Table 3). The minimum inhibitory concentration ranges of *P. macrocarpa* extracts (PMLE and PMBE) against the test organisms were from 0.125 to 2.55 mg/mL and those of *C. gigantea* extracts (CGLE and CGBE) were from 0.125 to 2.75 mg/mL (Table 2).

### 3.3. Anti-Inflammatory Activity

**Figure 5:**
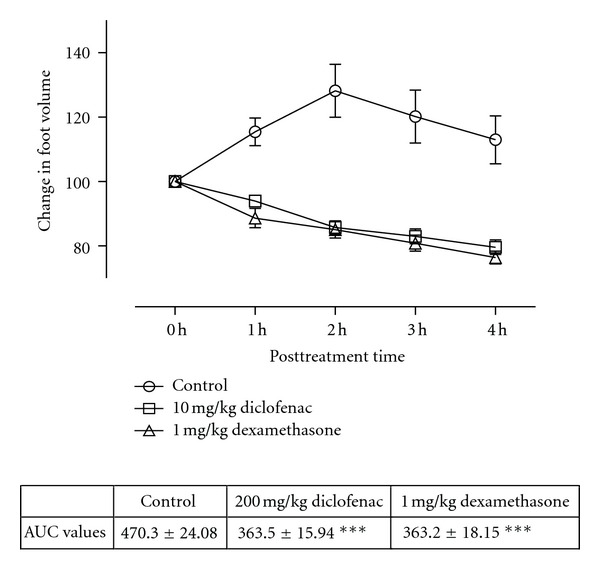
Effects of diclofenac (10 mg/kg) and dexamethasone (3 mg/kg) on carrageenan-induced paw oedema. Values are expressed as mean ± SEM (*N* = 5), significantly different from control. **P < 0.05, **P < 0.01, and ***P < 0.001. Control is the untreated birds.

All the animals injected with carrageenan exhibited acute inflammation which manifested as increased foot volume. The control group however showed increased inflammation till the fourth hour. All the extract-treated groups except CGLE- (*F*_3,80_ = 1.80, *P* = 0.1545: Figure 3) treated group exhibited significant anti-inflammatory effects (PMLE: *F*_3,80_ = 47.52, *P* < 0.0001; PMBE: *F*_3,80_ = 35.58, *P* < 0.001; CGBE: *F*_3,80_  =  1.80, *P* = 0.1545: Figures 1, 2, and 4). Similar effects were observed after treating the animals with diclofenac (*F*_3,80_ = 79.81, *P* < 0.0001) and dexamethasone (*F*_3,80_ = 90.49, *P* < 0.0001) used as positive controls (Figure 5).

## 4. Discussion

The present studies indicate the antimicrobial and anti-inflammatory properties of ethanol extracts of *P. macrocarpa *(PMLE and PMBE) and *C. gigantea* (CGLE and CGBE). These findings were similar to our previous work [27] with ethanol extract of *Funtumia elastica* Preuss Stapf. (Apocynaceae) which confirmed the plant as having both antimicrobial and anti-inflammatory properties. The antibacterial and antifungal activities of the *P. macrocarpa* are being reported for the first time. The MIC against the test bacteria is from the 0.125 to 2.55 mg/mL and the fungal agent (*C. albicans*) is from 0.75 to 1.75 mg/mL. With respect to the agar diffusion method, concentration of extracts from both leaves and the stem bark of *P. macrocarpa* less than 25 mg/mL did not exhibit activity against *S. aureus* and *P. aeruginosa* and less activity against *E. coli* at concentration of 25 mg/mL but the MICs for the previous test bacteria were comparable to the MICs of the *C. gigantea* extracts. This observation goes to support the assumption that size of inhibition halos of different extracts cannot be used for the determination of the relative antimicrobial potency since a more diffusible but less active extract could give a bigger diameter than a nondiffusible but more active extract [27, 31]. 

The antimicrobial activities exhibited by the ethanol extracts of leaves and stem bark of *C. gigantea* (CGLE and CGBE) are in line with previous antimicrobial works on the leaves of different species of *Cola* [22, 32, 33] where different extracts of cola were found to exhibit inhibitory activities against certain bacteria and fungi with respect to the leaf extract. The MIC range of both the leaf and stem bark extracts of *C. gigantea* against the test bacteria is from 0.125 to 2.75 mg/mL and the mean zones of inhibition of the different concentration (10–50 mg/mL) extracts (crude) were almost the same as those of the reference antibacterial agent, chloramphenicol at concentration of 1000 *μ*g/mL. However, with the agar diffusion technique, the leaf extracts of *C. gigantea* (10 to 50 mg/mL) showed more activity against both test bacteria and fungus compared to the extracts from the stem bark. *P. aeruginosa* was found to be generally less susceptible to all extracts from *P. macrocarpa* and *C. gigantean,* respectively (Table 3), and this was not surprising since it has been found to be resistant to most orthodox antibiotics [34]. The antimicrobial properties may justify the use of these plants for the treatment of various bacterial and fungal infections such as gonorrhea and urethral infections and sores.

The study also establishes the anti-inflammatory activity of the ethanol extracts of the leaves and stem bark of *P. macrocarpa* and *C. gigantean, *respectively. Carrageenan-induced oedema has been commonly used as an experimental animal model for acute inflammation and is established to be biphasic. The early phase (1 to 2 hours) of the carrageenan model is chiefly mediated by serotonin, histamine, and increased synthesis of prostaglandins in the damaged tissues. The late phase is sustained by prostaglandin release and mediated by bradykinin, leukotrienes, polymorphonuclear cells, and prostaglandins produced by tissue macrophages [35]. The extracts (PMLE, PMPE, and CGBE) inhibited the inflammation induced with carrageenan in both phases indicating the ability of these extracts to inhibit the synthesis or release of inflammatory mediators such as histamine, serotonin, bradykinin, and leukotrienes.

In comparing the four extracts (PMLE, CGLE, PMBE, and CGBE), only CGLE had insignificant anti-inflammatory activity in the carrageenan-induced hind paw oedema. However the CGBE had significant activity. The reason for this observation may be due to different chemical composition of the stem bark and leaves of the same plant. Traditionally, the bark is used in the management of haemorrhoids: dropsy, swelling, edema, gout, leprosy, and pain [15], and hence these results may confirm the medicinal uses of the bark. The CGBE extract may have relatively high amounts of the bioactive constituents in relation to the leaves. Phytochemical screening of *C. gigantea* showed the presence of alkaloids and this confirms the findings of Sonibare et al. [22]. Members of the cola family are closely related to Theobroma family of South America which have the methylxanthine alkaloids such as caffeine, theobromine, and theophylline as secondary metabolites. Caffeine is one of the alkaloids of genus cola and it is used as an analgesic and anti-inflammatory adjuvant [36, 37].

There was no significant difference in anti-inflammatory activity between the leaf and stem bark extracts (PMLE and PMBE) of *P. macrocarpa*. However, the leaves are traditionally used as diuretics, antiflatulence, and a remedy for stomach, bladder, and urinary problems [15].

Powdered dried leaves and stem bark of *P. macrocarpa *and *C. gigantea* showed the presence of tannins and with different tannin contents (1.02–1.57% w/w). Amoo and Agunbiade [38] reported on the high tannin content of the seeds of *P. macrocarpa*. Similar results were found in the samples of leaves and bark of *P. macrocarpa*. Tannins form a class of polyphenolic compounds which can act as antioxidants, antiviral, antibacterial, antiparasitic, and anti-inflammatory activity [39–42]. During the inflammatory cascade, antioxidants act as scavengers for free radicals protecting cells against oxidants which are mostly reactive oxygen and nitrogen species [43–45]. The aforementioned findings may justify the traditional medicinal uses of the *P. macrocarpa* and *C. gigantea* and hence there is the need to perform bioactivity fractionation of the active extracts to isolate the compounds that may be responsible for the antimicrobial and anti-inflammatory properties.

## 5. Conclusion

The minimum inhibitory concentration ranges of both ethanol leaf and bark extracts of *P. macrocarpa* against the test organisms were from 0.125 to 2.55 mg/mL and those of *C. gigantea* extracts were from 0.125 to 2.75 mg/mL. Extracts (10, 25, and 50 mg/mL) of *P. macrocarpa* and *C. gigantea* exhibited antimicrobial activity with concentrations of 50 mg/mL showing the highest zones of inhibition against the test organisms. All the extracts of *P. macrocarpa* and *C. gigantea* at 30, 100, and 300 mg/kg except leaf extract of *C. gigantea *exhibited significant anti-inflammatory activity. The aformentioned activities may confirm the ethnobotanical uses of these two plants as antimicrobial and anti-inflammatory agents. It is recommended that the bioactive extracts should be fractionated and the active compounds responsible for the previous pharmacological properties should be isolated.

## Figures and Tables

**Figure 1:**
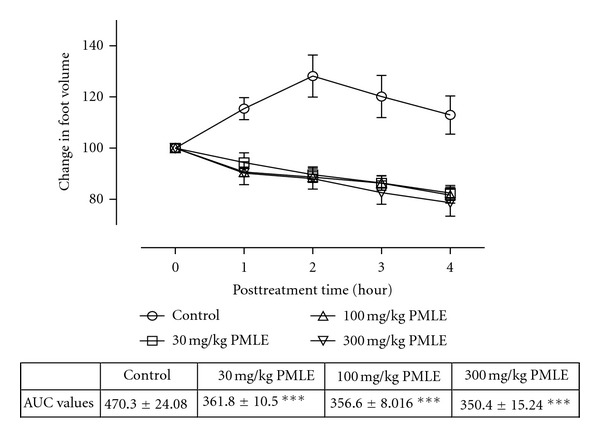
Effect of ethanol leaf extract (PMLE) of *P. macrocarpa *(30–300 mg/kg) on carrageenan-induced paw oedema. Values are expressed as mean ± SEM (*N* = 5), significantly different from control. **P < 0.05, **P < 0.01, and ***P < 0.001. Control is the untreated birds.

**Figure 2:**
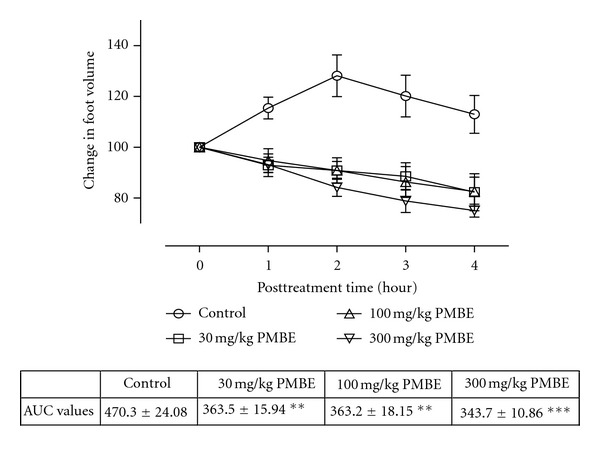
Effect of ethanol stem bark extract (PMBE) of *P. macrocarpa* (30–300 mg/kg) on carrageenan-induced paw oedema. Values are expressed as mean ± SEM (*N* = 5), significantly different from control. **P < 0.05, **P < 0.01, and ***P < 0.001. Control is the untreated birds.

**Figure 3:**
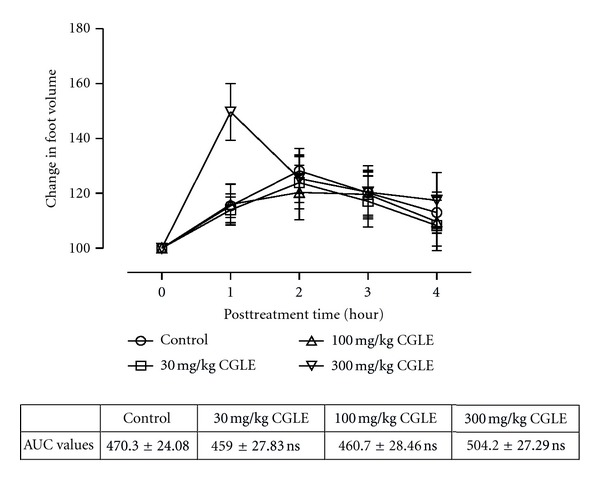
Effects of ethanol leaf extract (CGLE) of *C. gigantea* (30–300 mg/kg) on carrageenan-induced paw oedema. Values are expressed as mean ± SEM (*N* = 5), significantly different from control. *P < 0.05. Control is the untreated birds.

**Figure 4:**
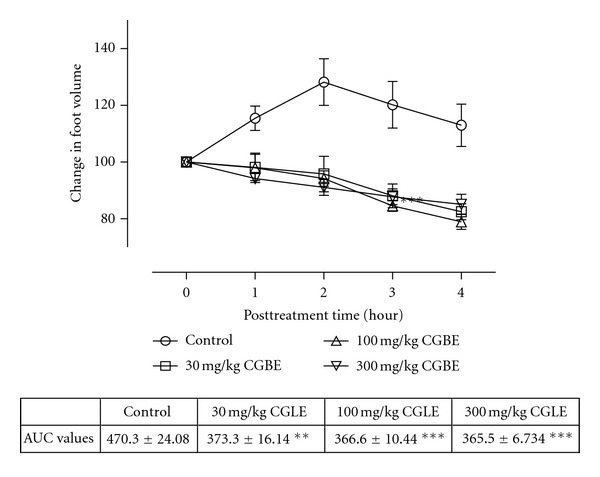
Effects of Ethanol stem bark extract (CGBE) of *C. gigantea* (30–300 mg/kg) on carrageenan-induced paw oedema. Values are expressed as mean ± SEM (*N* = 5), significantly different from control. **P < 0.05, **P < 0.01, and ***P < 0.001. Control is the untreated birds.

**Table 1:** Preliminary phytochemical screening of dried leaves and stem barks of *C. gigantea *(CG) and *P. macrocarpa *(PM). +: presence of secondary metabolite; −: absence of secondary metabolites.

Secondary metabolites	Alkaloids	Saponins	Flavonoids	Steroids	Carbohydrates	Sapogenetic glycosides	Tannins (% w/w)

Plant material/part							
CG leaf	+	+	+	+	+	−	1.57
CG stem bark	+	+	−	+	+	−	1.02
PM leaf	+	+	+	+	+	+	1.45
PM stem bark	+	+	−	+	+	+	1.14

**Table 2:** Minimum inhibitory concentrations (MICs) of ethanol leaf extract (CGLE) and ethanol stem bark extract (CGBE) of *C. gigantea *and ethanol leaf extract (PMLE) and ethanol stem bark extract (PMBE) of *P. macrocarpa *determined by microdilution method. The experiments were repeated three times. Reference antimicrobial agents: CPC: chloramphenicol; CTZ: clotrimazole; ND: Not determined.

Extract/MIC (mg/mL)	*S. aureus * ATCC 25923	*B. subtilis * NCTC 10073	*E. coli * ATCC 25922	*P. aeruginosa * ATCC 27853	*C. albicans*
CGLE	0.250	0.125	0.175	1.55	1.55
CGBE	0.125	0.150	0.250	2.75	1.75
PMLE	0.150	0.125	1.250	1.85	1.75
PMBE	0.125	0.175	0.155	2.55	0.75
CPC	0.025	0.020	0.025	0.055	ND
CTZ	ND	ND	ND	ND	0.025

**Table 3:** Antimicrobial activity of ethanol leaf extract (CGLE) and ethanol stem bark extract (CGBE) of *C. gigantea *and ethanol leaf extract (PMLE) and ethanol stem bark extract (PMBE) of *P. macrocarpa *by agar diffusion method. Activity of the methanol (solvent) used to dissolve the extracts was determined. Mean zones of inhibition (plus diameter of well) are mean (mm) of 3 independent experiments, mean ± SD, *n* = 4 replicates, and diameter of well/cup = 10 mm. Reference antimicrobial agents: CPC: chloramphenicol (1 mg/mL); CTZ: clotrimazole (1 mg/mL); ND: Not determined.

	Mean zones of growth inhibition (mm)				

Extract (mg/mL)	Test organisms				
	*S. aureus*	*B. subtilis*	*E. coli*	*P. aeruginosa*	*C. albicans*
	ATCC 25923	NCTC 10073	ATCC 25922	ATCC 27853	
CGLE					
10	19.67 ± 0.58	16.80 ± 0.40	12.20 ± 0.17	12.24 ± 0.13	12.65 ± 0.58
25	24.60 ± 0.53	19.33 ± 0.58	16.53 ± 0.24	15.50 ± 0.58	16.47 ± 0.48
50	28.33 ± 0.58	22.30 ± 0.42	19.67 ± 0.56	17.50 ± 0.47	22.13 ± 0.23
CGBE					
10	16.67 ± 0.58	13.33 ± 0.58	12.30 ± 0.58	0.0	13.67 ± 0.58
25	13.00 ± 0.00	17.80 ± 0.35	14.12 ± 0.42	0.0	18.90 ± 0.48
50	18.67 ± 0.58	19.50 ± 0.50	18.67 ± 0.58	13.47 ± 0.58	21.85 ± 0.23
PMLE					
10	0.0	17.80 ± 0.35	0.0	0.0	12.80 ± 0.20
25	14.60 ± 0.58	18.50 ± 0.50	19.33 ± 0.58	0.0	14.00 ± 0.00
50	19.67 ± 0.58	22.53 ± 0.12	21.47 ± 0.50	16.30 ± 0.23	22.33 ± 0.58
PMBE					
10	0.0	12.33 ± 0.58	11.33 ± 0.58	0.0	12.80 ± 0.20
25	14.50 ± 0.50	14.67 ± 0.58	12.67 ± 0.58	0.0	15.40 ± 0.36
50	17.13 ± 0.23	16.73 ± 0.58	15.83 ± 0.29	12.3 ± 1.20	18.60 ± 0.58
CPC	25.67 ± 0.38	31.21 ± 0.38	30.67 ± 0.85	22.33 ± 0.38	ND
CTZ	ND	ND	ND	ND	25.60 ± 0.61
Methanol	0.0	0.0	0.0	0.0	0.0
